# Diarrhœa of Teething Children

**Published:** 1873-11

**Authors:** W. H. Day


### ARTICLE X.

### *The Diarrhœa of Teething Children.*

BY DR. W. H. DAY.

The treatment of diarrhœa in teething children is apt to be looked at from a one-sided point of view; the quickest way to arrest it. We have diarrhœa, 1, from dental irritation; 2, from indigestion caused by over and under feeding; 3, from atmospheric changes. Then, too, the diarrhœa may be of a simple inflammatory, choleraic, or dysenteric character; each variety demanding a different plan of treatment.

Astringents, as a rule, are to be condemned. The diarrhœa will continue in spite of them, unless other precautions are taken. If the motions contain mucus and are slimy, and there is a trace of blood and redness about the anus, chalk mixture and kino will be of no service, nor will bismuth, acids, or oxide of zinc. The diet is primarily at fault in these cases, and undigested food has passed into the bowels. Warmth and complete rest, with a dose of castor oil in such cases, is the most appropriate treatment, though the gums may require puncturing, and a grain each of hydrargyrum cum creta and Dover's powder may be necessary. Occasionally a quarter grain of calomel, with a grain of Dover's powder, will be found of great value. Among hospital patients a large number of cases of diarrhœa are attributable to over suckling, and suckling by mothers in delicate health. The return of the catamenia is no hindrance to their nursing, and even menorrhagia in a mild or severe form. Remove all children suffering from diarrhœa from the breast, and let them have cow's milk diluted with lime water, previously warmed and given in a well rinsed bottle, and you will cure the diarrhœa.

Many children are reared entirely on Swiss milk, and this will now and then agree far better than cow's milk. Sometimes milk, in any form and however pure, will keep up the diarrhœa, and then cold barley water, or cold water thickened with isinglass will be necessary, or thin water arrowroot, to which a few drops of brandy may be added should the child be exhausted. Sometimes a powder containing two or three grains of rhubarb and carbonate of soda will neutralize the acidity which has resulted from the fermentative products of digestion, and set the little patients right with magical quickness. If the evacuations are free from mucus and blood, and there is no pain, a mild mixture of sulphate of magnesia and tincture of rhubarb may be prescribed in some cases with advantage. A drop of ipecacuanha wine in plain water, or mucilage and water, has been recommended, and it will often succeed.

Children are liable to diarrhœa at a certain season of the year from heat, and the excitement of travelling, and change from healthy country places or the seaside to the contaminated air of London.—*Brit. Med. Jour.*

